# Robotic Surgery in Acute Care: A Systematic Review of Its Role in Visceral Trauma and Emergency General Surgery

**DOI:** 10.7759/cureus.110954

**Published:** 2026-06-16

**Authors:** Vaibhav Jaiswal, Saumya Singh, CR Selvasekar, Subhash Khanna

**Affiliations:** 1 Trauma Surgery, King George's Medical University, Lucknow, IND; 2 Surgery, King George's Medical University, Lucknow, IND; 3 Surgery, The Christie NHS Foundation Trust, Manchester, GBR; 4 Gastrointestinal Surgery, GSL Medical College, Rajahmundry, IND; 5 Minimal Access, Gastrointestinal, and Robotic Surgery, Swagat Super Speciality Surgical Institute, Guwahati, IND

**Keywords:** acute care surgery and trauma, emergency general surgery, robotic-assisted surgery, robotic surgery, trauma surgery

## Abstract

Emergency laparotomies carry profound physiological morbidity, yet the adoption of minimally invasive surgery (MIS) in acute care remains hindered by laparoscopic limitations. While robotic platforms offer 3D visualization and wristed articulation to overcome these barriers, their application in emergency general surgery (EGS) and visceral trauma remains controversial. This systematic review aims to define the clinical and physiological boundaries of robotic surgery in the acute care setting.

A PRISMA-compliant systematic review was conducted using PubMed/MEDLINE, Embase, Scopus, the Cochrane Library, and Web of Science from database inception through March 2026. Eligible studies included adult patients undergoing robotic surgery for EGS or visceral trauma. To minimize analytical confounding, studies were synthesized within two predefined cohorts: EGS and visceral trauma. Primary outcomes included conversion to open surgery, technical success, and timing of intervention. Secondary outcomes included operative time, length of hospital stay, postoperative morbidity, and cost-related outcomes.

Thirty-six primary studies met the inclusion criteria, comprising 22 EGS studies and 14 visceral trauma studies. Within the EGS cohort, several comparative studies reported lower conversion-to-open rates with robotic surgery (0.0%-11.5%) compared with conventional laparoscopy (0.0%-28.7%), particularly in acute cholecystitis and complex hernia repair. Operative times were generally longer in robotic procedures, reflecting platform setup and docking requirements, whereas some studies reported shorter postoperative hospital stays. The visceral trauma literature consisted predominantly of case reports, case series, and observational studies. Robotic intervention was almost exclusively performed in hemodynamically stable patients during a delayed or semi-acute phase of care, with a median reported intervention interval of approximately 76 hours in the largest registry analysis. Reported applications were concentrated in complex diaphragmatic, thoracic, pancreatic, and pelvic reconstructions, with high rates of successful completion via minimally invasive techniques.

Current evidence suggests that robotic surgery is a feasible minimally invasive option in selected acute care scenarios. In EGS, robotic platforms may facilitate completion of complex procedures while maintaining low conversion rates in appropriately selected patients. In visceral trauma, robotic surgery appears most applicable during delayed or semi-acute reconstruction following physiological stabilization rather than during damage-control interventions. Further prospective studies are required to define patient selection criteria, clinical effectiveness, and cost-effectiveness in acute care surgery.

## Introduction and background

Trauma and emergency general surgery (EGS) constitute a major component of the global surgical burden [1]. Emergency operations account for approximately one-fifth of all surgical procedures worldwide and encompass a broad spectrum of time-sensitive conditions, including visceral trauma, perforation peritonitis, intestinal obstruction, strangulated hernias, and acute biliary disease [1,2]. Despite advances in perioperative care, emergency laparotomy remains associated with substantial morbidity and mortality, largely reflecting the physiological derangement, contamination burden, and inflammatory response that frequently accompany acute surgical disease [1,2].

A fundamental principle of acute care surgery is the distinction between patients who require immediate damage control surgery and those who can undergo definitive operative management. Damage control laparotomy (DCL) is a staged surgical strategy used in critically ill trauma patients with severe hemorrhage, contamination, or physiological instability, where the priority is rapid control of life-threatening pathology rather than definitive repair. However, only a minority of patients presenting with trauma or acute surgical pathology ultimately require damage-control intervention [1]. Many are hemodynamically stable or can be adequately resuscitated to permit definitive surgical treatment.

Minimally invasive surgery (MIS) has transformed elective surgical practice by reducing postoperative pain, shortening hospital stays, and minimizing long-term structural morbidity such as incisional hernias [3]. Nevertheless, adoption of MIS in trauma and emergency surgery has been comparatively slower because acute pathology often presents with inflammation, contamination, distorted anatomy, and time-sensitive decision-making [1,4]. These challenges frequently result in conversion to open surgery or the selection of an open approach from the outset.

Robotic surgical platforms represent an evolution of minimally invasive technology, providing three-dimensional visualization, tremor filtration, and articulated instrumentation that may facilitate dissection and reconstruction in anatomically confined operative fields [4,5]. However, the role of robotics in acute care surgery remains incompletely defined. Current evidence is largely derived from retrospective studies, registry analyses, and case reports, while concerns regarding operative duration, learning curves, resource utilization, and patient selection continue to limit widespread adoption [4-6].

Although robotic surgery is well established in several elective surgical specialties [7], its application in visceral trauma and EGS remains heterogeneous and has not been comprehensively evaluated within a unified acute care framework. Existing literature frequently combines elective and emergency cases, incorporates non-visceral specialties, or focuses on technical feasibility without clearly defining clinical indications and limitations [8].

Accordingly, the objective of this systematic review was to evaluate the current evidence regarding robotic surgery in visceral trauma and EGS. Specifically, we sought to examine its clinical applications, reported outcomes, technical feasibility, and implementation challenges, and to define the circumstances in which robotic platforms may have a role in contemporary acute care surgery.

## Review

Methods

Study Design and Protocol

This systematic review was conducted and reported in accordance with the Preferred Reporting Items for Systematic Reviews and Meta-Analyses (PRISMA) 2020 guidelines [9]. The objective was to evaluate the clinical outcomes, technical feasibility, and implementation challenges associated with robotic-assisted surgery in acute care settings. Because EGS and visceral trauma represent distinct clinical entities with differing pathophysiology, operative priorities, and levels of available evidence, studies pertaining to these domains were identified through a common search strategy but analyzed separately. A predefined review protocol specified independent synthesis of the EGS and visceral trauma cohorts to minimize clinical heterogeneity and avoid analytical confounding. Owing to substantial variation in study designs, patient populations, interventions, and outcome reporting, a quantitative meta-analysis was not considered appropriate; therefore, findings were synthesized descriptively across predefined outcome domains.

Literature Search Strategy

A comprehensive systematic literature search was performed across PubMed/MEDLINE, Embase, Scopus, the Cochrane Library, and Web of Science from database inception through March 2026. The search strategy combined controlled vocabulary terms (Medical Subject Headings (MeSH) in MEDLINE and Emtree terms in Embase) with free-text keywords related to robotic surgery, emergency general surgery, acute care surgery, and visceral trauma. Searches were restricted to studies published in the English language, and no date filters were applied. Database-specific search strategies were adapted to each platform's indexing structure and search syntax. The complete search strategies, including database-specific search strings, search dates, and applied filters, are provided in Supplementary Appendix 1.

Intervention terms: "Robotic surgery," "robot-assisted," "robotic surgical procedures," "da Vinci," "surgical robotics."

Condition terms: "Emergency general surgery," "acute care surgery," "visceral trauma," "abdominal trauma," "acute cholecystitis," "incarcerated hernia," "perforated viscus," "damage control laparotomy."

Boolean operators (AND, OR) were applied to optimize search sensitivity, and truncation filters were utilized to capture all root-word variations. Following the primary database search, the reference lists of all included articles and relevant narrative reviews were manually cross-referenced to identify any additional eligible studies that eluded the initial algorithmic sweep.

Eligibility Criteria (PICO Framework)

Study inclusion and exclusion criteria were defined strictly using the PICO (Population, Intervention, Comparison, and Outcomes) framework.

Population (P): Adult patients (>18 years) presenting with either acute non-trauma surgical emergencies (e.g., acute cholecystitis, incarcerated/strangulated hernias, gastrointestinal perforations, or obstructions) OR complex visceral trauma (blunt or penetrating thoracoabdominal injuries) requiring urgent, emergent, or semi-acute surgical intervention.

Intervention (I): Utilization of a robotic-assisted surgical platform (e.g., da Vinci Surgical System and CMR Versius) as the primary or attempted modality for exploration, resection, or definitive repair.

Comparison (C): Where applicable (primarily in the EGS cohort), the comparison group consisted of patients undergoing conventional laparoscopic or open surgical approaches for identical acute pathologies. Non-comparative case series were permitted in the visceral trauma cohort to evaluate early feasibility.

Outcomes (O): Primary outcomes included conversion rate to open laparotomy/thoracotomy, technical success of the repair, and the exact timing of the intervention (delay from admission). Secondary outcomes included total operative time (to quantify the "docking penalty"), hospital length of stay (LOS), postoperative morbidity (specifically surgical site infections (SSIs) and systemic inflammatory response), and economic cost-utility.

Exclusion Criteria

Studies were excluded if they evaluated elective surgeries, pediatric populations (<18 years), purely non-clinical parameters (e.g., ex vivo simulation models), or if the data failed to distinguish emergency from elective outcomes. Articles not available in English or lacking full-text availability were also excluded.

Study Selection and Data Extraction

**Figure 1:**
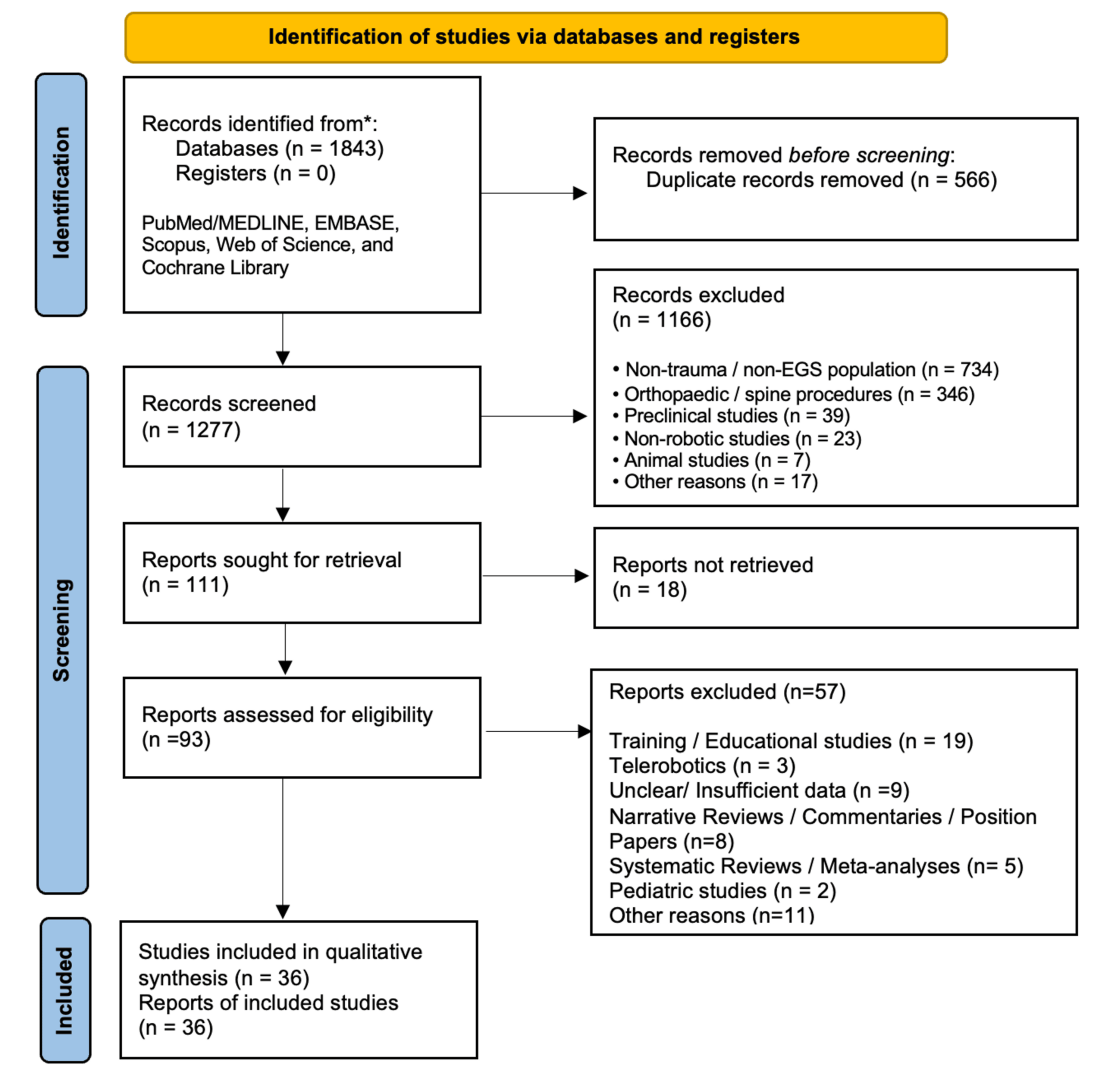
PRISMA flow diagram for study selection. This PRISMA 2020 [9] flow diagram summarizes the study selection process. Following database screening and full-text eligibility assessment, a secondary methodological review was undertaken to ensure that only primary empirical studies contributed to the final outcome synthesis. Consequently, narrative reviews, commentaries, position papers, systematic reviews, and meta-analyses were excluded from the analytical dataset. Pediatric trauma studies identified during the secondary review were also excluded to maintain strict adherence to the predefined adult-only inclusion criteria. The final qualitative synthesis comprised 36 primary studies, including 22 emergency general surgery (EGS) studies and 14 visceral trauma studies.

Two independent reviewers screened all retrieved titles and abstracts for relevance after duplicates were removed. The full texts of potentially eligible articles were subsequently reviewed against the strict PICO criteria. Any discrepancies between the two reviewers regarding study inclusion were resolved via consensus or consultation with a senior third reviewer. The selection process and exact attrition of records are detailed in the PRISMA flow diagram (Figure 1).

Data extraction was performed using standardized, pre-formatted spreadsheets. Extracted variables included study characteristics (author, year, design, evidence level), patient demographics, specific anatomical pathology or injury zone, timing of intervention, and predefined primary and secondary clinical outcomes.

Quality Assessment, Risk of Bias, and Exclusion Verification

Methodological quality and risk of bias were independently assessed by two reviewers. Comparative observational studies within the EGS cohort were evaluated using the Newcastle-Ottawa Scale (NOS) [10]. As the visceral trauma literature consisted predominantly of case reports and case series, these studies were appraised using the Joanna Briggs Institute (JBI) critical appraisal checklists for case reports and case series, as appropriate [11]. Quality assessment results are provided in Supplementary Appendix 2.

To ensure the integrity of the final dataset and strict adherence to the predefined eligibility criteria, all included studies underwent secondary verification following full-text review. Particular attention was directed toward the adult-only inclusion criterion. Studies identified as involving pediatric populations or lacking sufficient primary clinical data were excluded from the final synthesis. This process resulted in the exclusion of two pediatric trauma reports identified during the secondary review process.

Bibliometric Trend Analysis

**Figure 2:**
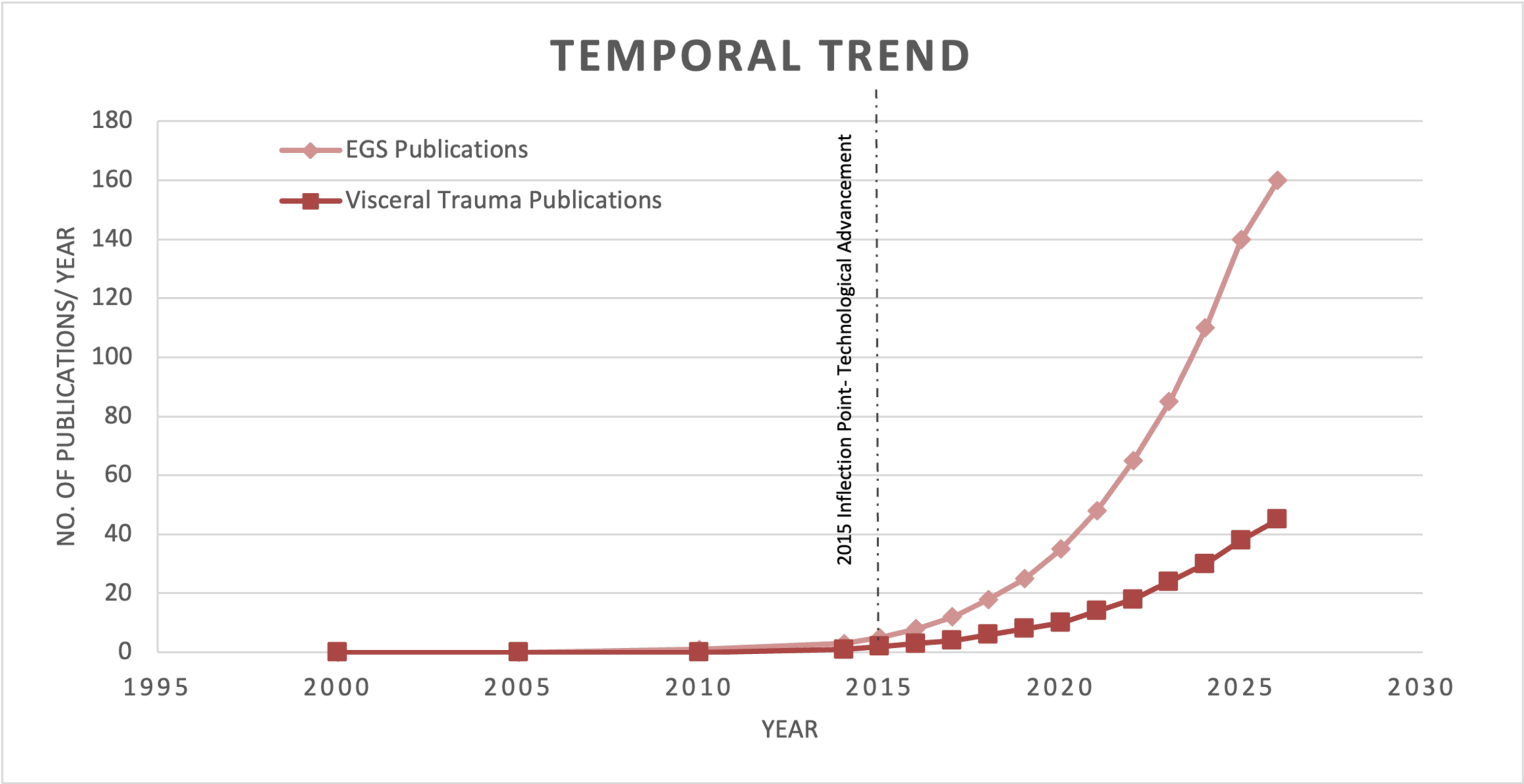
Temporal trends in robotic acute care research: comparative growth in EGS and visceral trauma (2000-2026). This graph illustrates the volume of peer-reviewed publications and records identified over a 26-year period. A notable technological inflection point was observed in 2015, triggering exponential growth in both fields. While research in emergency general surgery (EGS) has surged to 160 publications annually by 2026, visceral trauma research shows a more conservative trajectory (45 publications), reflecting the clinical and physiological constraints of robotic application in acute hemorrhage and damage control scenarios.

To contextualize the evolution of robotic surgery within acute care, a supplementary descriptive bibliometric trend analysis was performed (Figure 2). The PubMed/MEDLINE database was queried using broad MeSH terms and associated keywords pertaining to robotic surgery, emergency general surgery, and visceral trauma. Annual publication counts were extracted from January 2000 through May 2026 and categorized according to EGS and visceral trauma themes. Following the removal of duplicate records, publication frequencies were plotted chronologically to illustrate changes in research activity over time. This analysis was undertaken solely to provide historical and contextual insight into the growth of the field and was not intended to constitute a formal bibliometric, scientometric, citation-network, or impact analysis.

Data Synthesis

Given the substantial clinical and methodological heterogeneity among the included studies, formal quantitative meta-analysis was not considered appropriate. Variations in study design, patient populations, emergency pathologies, operative techniques, and outcome reporting precluded meaningful statistical pooling. Consequently, findings were synthesized descriptively according to predefined outcome domains.

To minimize analytical confounding, studies were evaluated within two predefined cohorts: EGS and visceral trauma. For the EGS cohort, outcomes including conversion to open surgery, operative time, length of hospital stay, postoperative morbidity, and cost were summarized descriptively from the available primary studies. For the visceral trauma cohort, where evidence consisted predominantly of case reports and small case series, synthesis focused on patterns of clinical application, timing of intervention, anatomical indications, technical feasibility, and reported outcomes.

Results

Literature Search and Cohort Bifurcation

Following PRISMA-guided study selection, 36 studies met the final inclusion criteria (Figure 1). To minimize analytical confounding between two distinct acute care populations, the included literature was stratified into EGS and visceral trauma cohorts. The EGS cohort comprised 33 studies, of which 22 primary empirical studies contributed to outcome synthesis, while 11 narrative reviews, commentaries, meta-analyses, and position statements were retained for contextual interpretation. The visceral trauma cohort comprised 14 studies evaluating the feasibility, timing, technical application, and clinical outcomes of robotic surgery in adult trauma patients. Given the substantial clinical and methodological heterogeneity across both cohorts, findings were synthesized descriptively according to predefined outcome domains.

Emergency General Surgery (EGS) Cohort

Following secondary methodological review, 11 non-primary publications, including narrative reviews, commentaries, meta-analyses, and position statements, were excluded from outcome synthesis. Consequently, the final EGS cohort comprised 22 primary studies, including retrospective cohort studies, registry analyses, administrative database investigations, propensity-matched studies, case-matched studies, feasibility studies, and case series, representing predominantly level II-III evidence. As EGS encompasses a heterogeneous spectrum of biliary, hernia, colorectal, and foregut pathologies with differing anatomical, physiological, and operative characteristics, formal statistical pooling was considered inappropriate. Outcomes were therefore synthesized descriptively by disease-specific clinical context. The characteristics of the included studies and their principal findings relevant to the predefined review outcomes are summarized in Table 1.

**Table 1:** Characteristics and principal findings of primary studies included in the emergency general surgery (EGS) cohort (n = 22). The table summarizes the 22 primary studies included in the emergency general surgery (EGS) cohort following secondary methodological review. Studies are categorized according to design, target pathology, sample size, and principal findings relevant to the predefined review outcomes, including technical feasibility, conversion to open surgery, operative duration, postoperative morbidity, length of hospital stay, and cost. Owing to substantial clinical and methodological heterogeneity among included pathologies, the table is intended to provide a descriptive overview of the available evidence rather than a pooled quantitative analysis. HCUP-SID: Healthcare Cost and Utilization Project - State Inpatient Databases; Florida AHA: Florida Agency for Healthcare Administration; ACS-NSQIP: American College of Surgeons National Surgical Quality Improvement Program; ACHQC: Abdominal Core Health Quality Collaborative; RACSP: robotic acute care surgery program; LOS: length of stay; MIS: minimally invasive surgery; AAST: American Association for the Surgery of Trauma; IPTW: inverse probability treatment weighting.

Study (year)	Study design	Target pathology	N	Key intended outcome/Findings
Mahmoud et al. [12]	Retrospective cohort	Interval cholecystectomy	215	0% robotic conversion vs. 19% laparoscopic; lower estimated blood loss.
Pather et al. [13]	Retrospective cohort	Paraesophageal hernia	207	Urgent robotic repair proven as safe as elective repair with equivalent LOS.
McMahon et al. [14]	Case report	Fecopneumothorax	1	Highlighted the severe morbidity of delayed complex hernia repairs.
Kim & Towfigh [15]	Registry (ACHQC)	Ventral hernia	73,241	Identified patient acuity/complexity drives outcomes more than surgical specialty.
Maertens et al. [16]	Case series	Colorectal emergencies	10	Zero conversions; 9.4-day median LOS during pandemic limitations.
Jecius et al. [17]	Database (ACS-NSQIP)	Emergent colectomy	1,855	MIS approaches (including robotic) yield lower complication and mortality rates.
Robinson et al. [18]	Retrospective cohort	Perforated ulcer	44	Faster in-room start times for robotic; zero open conversions.
Hosein et al. [19]	Database (Vizient)	Hiatal hernia	9,171	Demonstrated superiority of minimally invasive approaches over open in urgent settings.
Ceccarelli et al. [20]	Case series	Giant hiatal hernia	5	Proved technical feasibility of resolving strangulated hernias via MIS.
Ricciardiello et al. [21]	Retrospective cohort	Cholecystitis	27	Validated feasibility and cosmetic outcomes of single-site robotic EGS.
Sugiyama et al. [22]	Retrospective cohort	Cholecystitis	592	Robotic outcomes equivalent to laparoscopy across all AAST severity grades.
Grimsley et al. [23]	Database (Florida AHA)	General EGS	60,733	Propensity-matched analysis showing higher costs but comparable outcomes to laparoscopy.
Kudsi et al. [24]	Propensity-matched	Ventral hernia	526	Proved the safety and feasibility of robotic repair in patients with BMI > 35.
Nzenwa et al. [25]	Retrospective cohort	Cholecystitis	3,179	Robotic-assisted interval approach is non-inferior to laparoscopy but incurs higher costs.
Greenberg et al. [26]	Database analysis	Cholecystitis	29,937	IPTW-matched analysis showing reduced odds of open conversion but increased subtotal cholecystectomies.
Cole et al. [27]	Case-matched	General EGS	369	Safety validated during the 'adoption phase' of robotic emergency care.
Bou-Ayash et al. [28]	Feasibility study	Inguinal hernia	23	Short LOS (1.4 days) and extremely low complication profile for incarcerated hernias.
Janjua et al. [29]	Database (HCUP-SID)	Inguinal hernia	36,396	The robotic approach had the lowest LOS but the highest inpatient cost.
Presl et al. [30]	Retrospective analysis	Sigmoid colectomy	83	Demonstrated reduced operative trauma and comparable cost-effectiveness.
Klein et al. [31]	Retrospective cohort	Cholecystitis	260	Proved clinical benefit and shorter operative times for grade B and C cholecystitis.
Jose et al. [32]	Institutional cohort	RACSP implementation	156	Institutional protocol decreased open cholecystectomy and hernia rates significantly.
Kubat et al. [33]	Retrospective cohort	Cholecystectomy	150	Urgent operative times significantly longer than elective due to the learning curve.

Acute Biliary Pathologies

**Figure 3:**
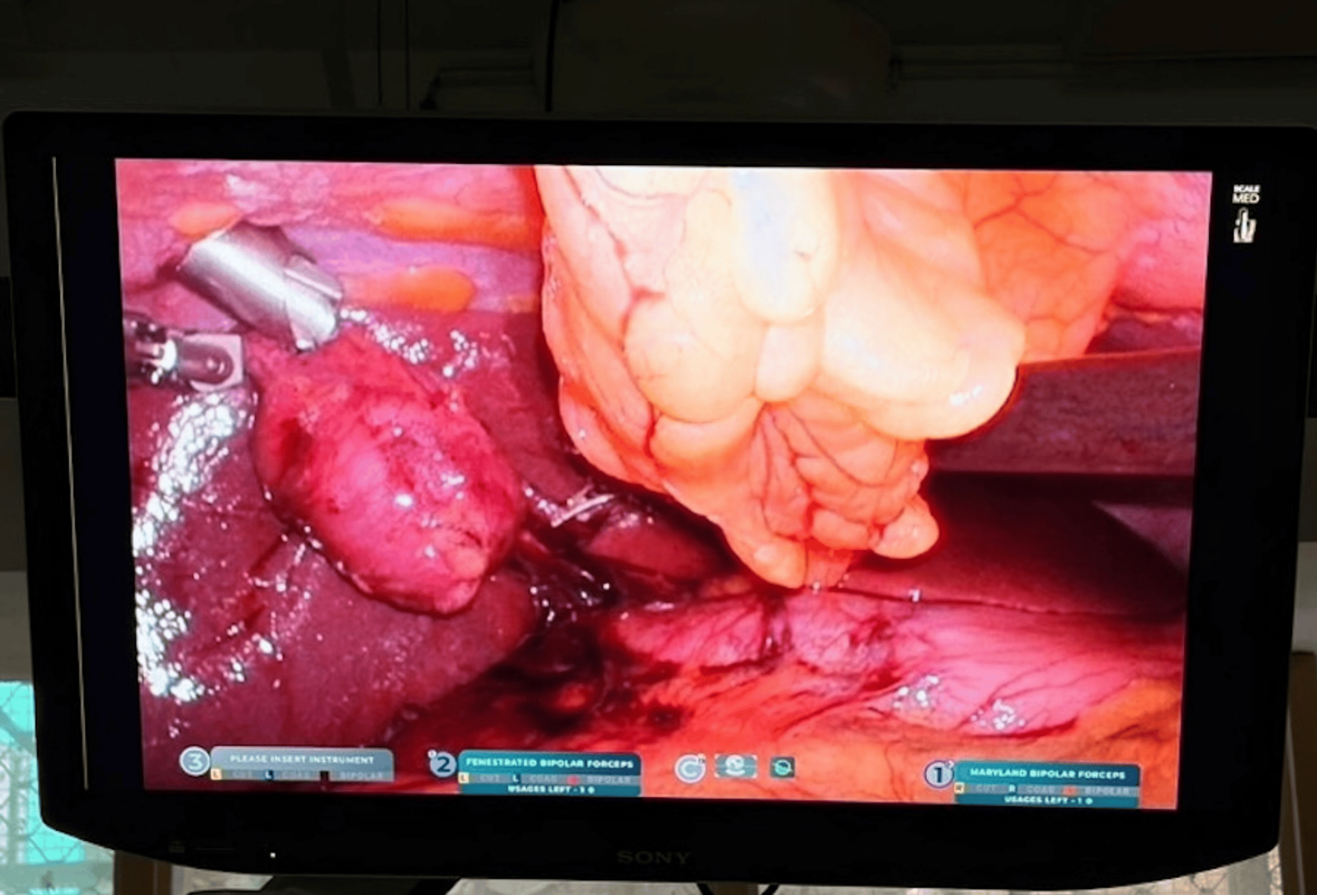
External high-definition vision monitor display showcasing active robotic dissection in acute cholecystitis.

Seven studies evaluated robotic approaches in acute cholecystitis and interval cholecystectomy [12,21,22,25,26,31,33]. Across these studies, robotic cholecystectomy demonstrated high technical feasibility with generally low reported conversion-to-open rates. In interval cholecystectomy following gallbladder drainage procedures, robotic surgery achieved a 0% conversion rate compared with 19% for conventional laparoscopy [12]. A matched database analysis of 29,937 patients reported lower odds of conversion to open surgery among robotic cases, although subtotal cholecystectomy was performed more frequently [26]. In contrast, a multicenter cohort evaluating 592 patients across all American Association for the Surgery of Trauma (AAST) severity grades found comparable conversion rates and intraoperative outcomes between robotic and laparoscopic approaches [22]. The external high-definition vision monitor provides the entire operating team with real-time, stereoscopic visualization of the inflamed, edematous gallbladder, while the integrated dashboard tracks active console instrument status throughout the dissection (Figure 3). Collectively, the available evidence supports the feasibility of robotic cholecystectomy in selected emergency settings, although outcome reporting remained heterogeneous across studies.

Hernia and Foregut Emergencies

Seven studies evaluated robotic surgery for paraesophageal, hiatal, ventral, and inguinal hernias [13,15,19,20,24,28,29]. Urgent robotic paraesophageal hernia repair achieved perioperative outcomes comparable to elective repair without prolonging hospital stay [13]. Analysis of 9,171 hiatal hernia repairs demonstrated that, although open approaches were used three times as frequently in urgent settings, open repairs were associated with longer stays (9.4 days versus less than four days) and higher postoperative complication rates compared with laparoscopic and robotic cohorts [19]. Small case series and feasibility studies reported successful completion of emergency robotic hernia repairs with low complication rates and no reported conversions [20,28]. Registry-based analyses suggested that patient acuity and defect complexity were major determinants of outcome independent of operative platform [15]. Robotic ventral hernia repair was also reported to be feasible in obese patients and complex abdominal wall defects [24].

Colorectal Emergencies and Foregut Perforation

Five studies evaluated robotic surgery in emergent colorectal pathology and upper gastrointestinal perforation [16-18,30,32]. Analysis of 1,855 emergent colectomies for colorectal cancer using the American College of Surgeons National Surgical Quality Improvement Program (ACS-NSQIP) dataset found that conventional open laparotomies were associated with higher rates of 30-day mortality (6.7% versus 3.8%) and overall complications (28.1% versus 16.7%) compared to minimally invasive approaches [17]. A case series of emergency colorectal resections reported successful minimally invasive completion without conversion to laparotomy [16]. In sigmoid colectomy for diverticular emergencies, robotic surgery was associated with lower postoperative inflammatory marker levels and earlier return of bowel function [30]. Robotic repair of perforated gastrojejunal ulcers has also been reported to be technically feasible, with successful, minimally invasive completion in all cases [18].

Operative Duration

Operative time was reported variably across studies and reflected both disease complexity and institutional experience. Longer operative times were reported for urgent robotic cholecystectomy compared with elective procedures (95.0 versus 71.9 minutes), reflecting the technical challenges associated with inflamed acute pathology [33]. However, data from mature robotic programs suggest that the platform's wristed dexterity balances active dissection times as disease severity increases. A study at a level 1 trauma center found that robotic cholecystectomy resulted in shorter total operative times compared to conventional laparoscopy for moderate, grade B (101.8 minutes vs. 114.9 minutes) and severe, grade C (134.6 minutes vs. 152.0 minutes) biliary inflammation. Logistically, it was noted that institutional optimization can mitigate standard setup delays, with the robotic cohort reaching "in-room-to-surgery-start" faster than laparoscopy (25 minutes versus 31 minutes) [18]. Overall, operative duration findings were inconsistent and highly dependent on pathology, surgeon experience, and institutional workflow.

Length of Stay, Morbidity, and Cost

Hospital LOS was reported across multiple studies and generally ranged from short postoperative admissions following incarcerated hernia repair to longer hospitalizations associated with complex colorectal emergencies [17,20,24]. Analysis of 36,396 inpatient inguinal hernia repairs demonstrated that the robotic subset had a shorter inpatient stay (2.3 days) compared with laparoscopic (3.2 days) and open (4.2 days) techniques [29]. However, direct inpatient hospital costs for the robotic cohort were approximately 38% higher than those for the open and laparoscopic cohorts [29].

This cost premium was corroborated in a study of 3,179 matched advanced cholecystitis records, in which robotic interval cholecystectomies incurred higher hospital costs ($17,077 versus $13,531) despite comparable morbidity profiles [25]. Similarly, among 60,733 emergency surgeries, direct capital surgical expenses drove total costs higher for robotic cohorts across common acute general surgery procedures [23].

Postoperative morbidity was evaluated in several cohort and database studies. Lower complication rates were reported for minimally invasive colectomy compared with open surgery [17], while feasibility studies reported low SSI rates and acceptable perioperative safety profiles [28]. Across comparative studies, morbidity outcomes were generally comparable between robotic and laparoscopic approaches, although outcome definitions varied substantially between investigations.

Conversely, in highly complex, tissue-quantified hindgut emergencies, upfront instrumentation costs may be offset by attenuated tissue trauma. Presl et al. conducted an institutional analysis of sigmoid colectomies for diverticular emergencies and reported that the robotic approach was associated with a lower postoperative C-reactive protein spike and a faster return of bowel function (1.63 days versus 2.19 days) [30]. This accelerated clinical recovery translated into lower collective hospitalization charges per patient for the robotic approach compared with conventional laparoscopy [30].

Visceral Trauma Cohort

Following secondary methodological review, two pediatric case reports and one systematic review were excluded from outcome synthesis. Consequently, the final visceral trauma cohort comprised 14 primary studies, including one registry-based observational study and 13 case reports (Table 2). Compared with the EGS cohort, the trauma literature represents an early-stage evidence base characterized by highly selected patients, heterogeneous injury patterns, and predominantly descriptive reporting of outcomes. Accordingly, findings were synthesized according to timing of intervention, anatomical application, and reported technical outcomes.

**Table 2:** Characteristics and principal findings of primary studies included in the visceral trauma cohort (n = 14). The table summarizes the 14 primary studies included in the visceral trauma cohort following secondary methodological review and exclusion of pediatric reports and non-primary literature. Studies are categorized according to injury pattern, anatomical region, timing of robotic intervention, and principal findings relevant to the predefined review outcomes. The available evidence consists predominantly of case reports and small observational studies, reflecting the exploratory nature of robotic applications in trauma surgery. Owing to substantial heterogeneity in injury mechanisms, anatomical locations, and outcome reporting, the table is intended to provide a descriptive overview of the current evidence rather than a pooled quantitative analysis. ACS-TQIP: American College of Surgeons Trauma Quality Improvement Program.

Study	Design	Injury/Anatomical zone	N	Timing of robotic intervention	Principal outcome
Balthazar da Silveira et al. [34]	Retrospective cohort	Traumatic lateral abdominal wall hernia	21	Delayed reconstruction	Safe repair; 9.5% conversion
Gutierrez et al. [35]	Case report	Intraperitoneal bladder rupture	1	Day 4 after injury	Successful repair without conversion
Fitzgerald et al. [36]	Case report	Diaphragmatic intercostal hernia	1	18 months after injury	Successful mesh reconstruction
Todderud et al. [37]	Case report	Aortopulmonary bullet retrieval	1	Day 6	Successful extraction without vascular injury
Setia et al. [38]	Case report	Post-traumatic hydronephrosis	1	2 years after trauma	Successful robotic partial nephrectomy
Arda et al. [39]	ACS-TQIP registry	Multi-system trauma	210	Median 76 hours	Conversion 27.6%; mortality 3.3%
Holder et al. [40]	Case report	Traumatic diaphragmatic hernia	1	37 days	Successful reduction and primary repair
Nehme et al. [41]	Case report	Tracheal gunshot injury	1	Semi-acute	Successful robotic airway repair
Rollins et al. [42]	Case report	Grade IV splenic injury	1	Following failed embolization	Successful robotic splenectomy
Marshall et al. [43]	Case report	Bronchial disruption	1	Semi-acute	Successful robotic reconstruction
Counts et al. [44]	Case report	Right diaphragmatic rupture	1	Semi-acute	Successful transthoracic repair
Griffin et al. [45]	Case report	Extraperitoneal bladder injury	1	Delayed after instability	Successful robotic cystorrhaphy
Wang et al. [46]	Case report	Left main bronchial rupture	1	33 days	Successful robotic reconstruction
Kim et al. [47]	Case report	Right diaphragmatic rupture	1	Index admission (stable patient)	Successful robotic repair

Timing of Intervention and the "Semi-Acute" Window

A consistent finding across the included studies was that robotic surgery was not utilized during the initial damage-control phase of trauma management. Instead, robotic intervention was performed in hemodynamically stable patients following completion of resuscitation and physiological stabilization. The largest available dataset, derived from the American College of Surgeons Trauma Quality Improvement Program (ACS-TQIP) registry, reported a median interval of 76 hours between admission and robotic intervention [39]. Similarly, individual case reports described robotic procedures performed in delayed or semi-acute settings, ranging from several days after injury to reconstruction of late traumatic sequelae months or years after the initial event. Robotic intervention was also reported following failure of non-operative management strategies, including angioembolization for splenic trauma [42]. Collectively, the available evidence suggests that robotic trauma surgery is predominantly utilized as a delayed or secondary reconstructive modality rather than an emergency damage-control procedure.

Anatomical Distribution and Technical Applications

The reported applications of robotic surgery were concentrated within anatomically confined regions where conventional open exposure is associated with substantial operative morbidity. Frequently described indications included diaphragmatic injuries [36,40,44,47], tracheobronchial disruptions [41,43,46], genitourinary trauma [35,38,45], complex abdominal wall reconstruction [34], thoracic foreign-body retrieval [37], and delayed management of solid organ injury [42].

Several studies described successful completion of technically demanding reconstructive procedures, including primary diaphragmatic repair, bronchial reconstruction, tracheal repair, bladder reconstruction, partial nephrectomy, and complex abdominal wall repair. Notably, delayed robotic reconstruction of traumatic left main bronchial rupture was successfully performed 33 days after injury with satisfactory functional recovery [46]. Similarly, robotic transthoracic repair of right-sided diaphragmatic rupture was reported both during the index admission and in delayed presentations without the need for thoracotomy or laparotomy [44,47].

Clinical Outcomes

Clinical outcome reporting was heterogeneous and largely limited to descriptive data. The ACS-TQIP analysis reported a conversion-to-open rate of 27.6% and an overall mortality rate of 3.3% among carefully selected trauma patients undergoing robotic intervention [39]. Most case reports described successful minimally invasive completion of the intended procedure without major perioperative complications or mortality. However, interpretation of these findings remains limited by the predominance of single-patient reports, the absence of comparative studies, and substantial variation in injury severity, anatomical location, and timing of intervention. Consequently, the available literature primarily demonstrates technical feasibility in selected hemodynamically stable trauma patients rather than comparative effectiveness against conventional operative approaches.

Discussion

The Laparotomy Burden Versus the Robotic MIS Edge

The physiological burden of an emergency or trauma laparotomy is profound. Recent literature unequivocally demonstrates that open emergency laparotomies carry exponentially higher rates of SSIs, often 35-46% in contaminated fields, along with increased risks of fascial dehiscence and long-term incisional hernias compared with elective procedures [48]. While MIS effectively mitigates this massive "second hit," conventional laparoscopy has historically seen limited adoption in severe EGS and trauma [4]. Conventional laparoscopy is inherently limited by two-dimensional visualization, rigid instrumentation, and fulcrum-effect ergonomics. In the acute setting, these limitations become prohibitive when attempting to run dilated bowel in an obstruction or to safely dissect severely inflamed, gangrenous tissues. Robotics represents the advanced evolution of MIS. The integration of high-definition 3D visualization and wristed articulation overcomes the limitations of laparoscopy. It allows surgeons to operate in distorted emergency planes with the precision of an open laparotomy, but without the morbid fascial incision, directly mitigating the 40% SSI risk inherent to emergency open abdomens [4,49]. This technological advancement permits enhanced dexterity and range of motion, providing a critical advantage in emergent, technically challenging scenarios [50].

Pathology-Specific EGS Evidence: Translating Technical Ease to Clinical Benefit

Skeptics frequently argue that the robotic platform offers "technical ease" to the surgeon without delivering tangible clinical improvements for the patient. Our synthesis of the EGS data (n = 22 studies) directly refutes this, demonstrating that technical ease translates to patient safety across specific acute pathologies. In acute cholecystitis, wristed instruments enable safe dissection of Calot’s triangle even in severe, gangrenous inflammation, contributing to the robotic cohort's lower conversion rates (0.0-11.5%) compared with laparoscopy (0.0-28.7%) [26]. Furthermore, in emergency hernias and gastrointestinal perforations/obstructions, conventional laparoscopic suturing of a perforated viscus or closure of an emergency ventral defect under tension is highly demanding. The robotic platform facilitates robust, tension-free intracorporeal suturing and provides dynamic multi-quadrant reach to efficiently mobilize the bowel. Avoiding a laparotomy in these highly contaminated cases directly reduces postoperative systemic inflammatory response syndrome [6]. This advanced capability thereby ameliorates the inflammatory cascade often exacerbated by larger incisions and prolonged operative times, contributing to improved patient outcomes in emergent settings [4].

The Trauma Paradigm: Beyond Damage Control

In acute trauma, the established DCL paradigm prioritizes rapid control of hemorrhage and contamination over definitive repair [51]. Deploying a robotic platform during the initial DCL phase is generally inappropriate, as robotic trauma surgery has been reported almost exclusively in hemodynamically stable patients following completion of resuscitation. The largest registry analysis identified a median delay of 76 hours between admission and robotic intervention, supporting its current role as a delayed or semi-acute reconstructive modality rather than a damage-control tool [39]. Therefore, the application of robotics in trauma lies strictly outside the damage control paradigm. The current application of robotic surgery in trauma appears to be confined to a “semi-acute window” in hemodynamically stable patients, particularly for definitive secondary repairs requiring high precision, such as suturing diaphragmatic lacerations, complex solid-organ resections, or deep pelvic reconstructions, where open access is associated with significant morbidity [8]. This approach maximizes the benefits of robotic assistance in reconstructive phases, minimizing the physiological insult associated with further invasive procedures in recuperating trauma patients [52]. This strategic deployment of robotic techniques also aligns with the broader goal of reducing hospital LOS and accelerating recovery in complex trauma cases [53].

The Docking Penalty Versus the Conversion Trade-Off

**Figure 4:**
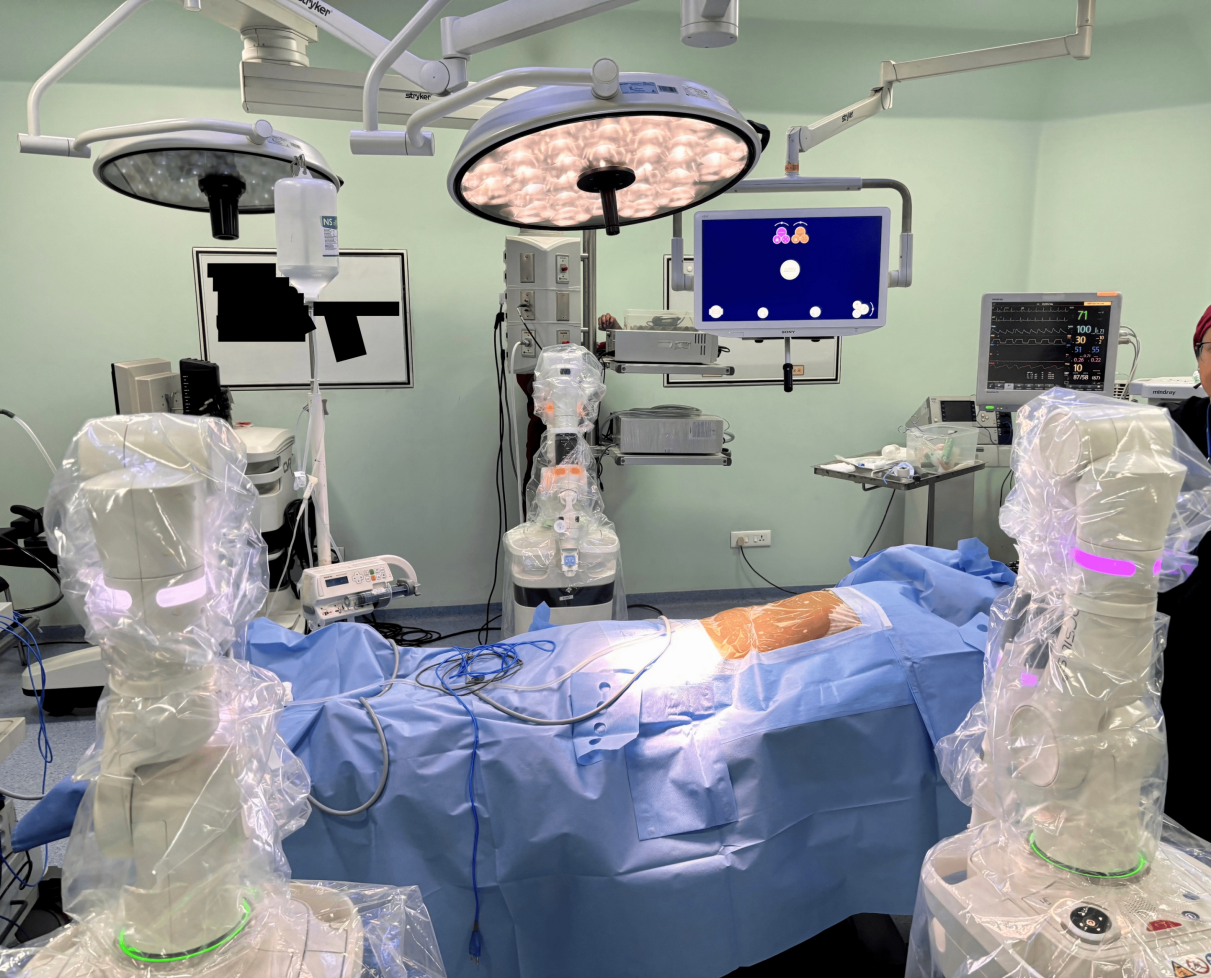
The global OR setup. Spatial configuration and modular operating room (OR) layout arranged to ensure rapid emergency undocking access.

**Figure 5:**
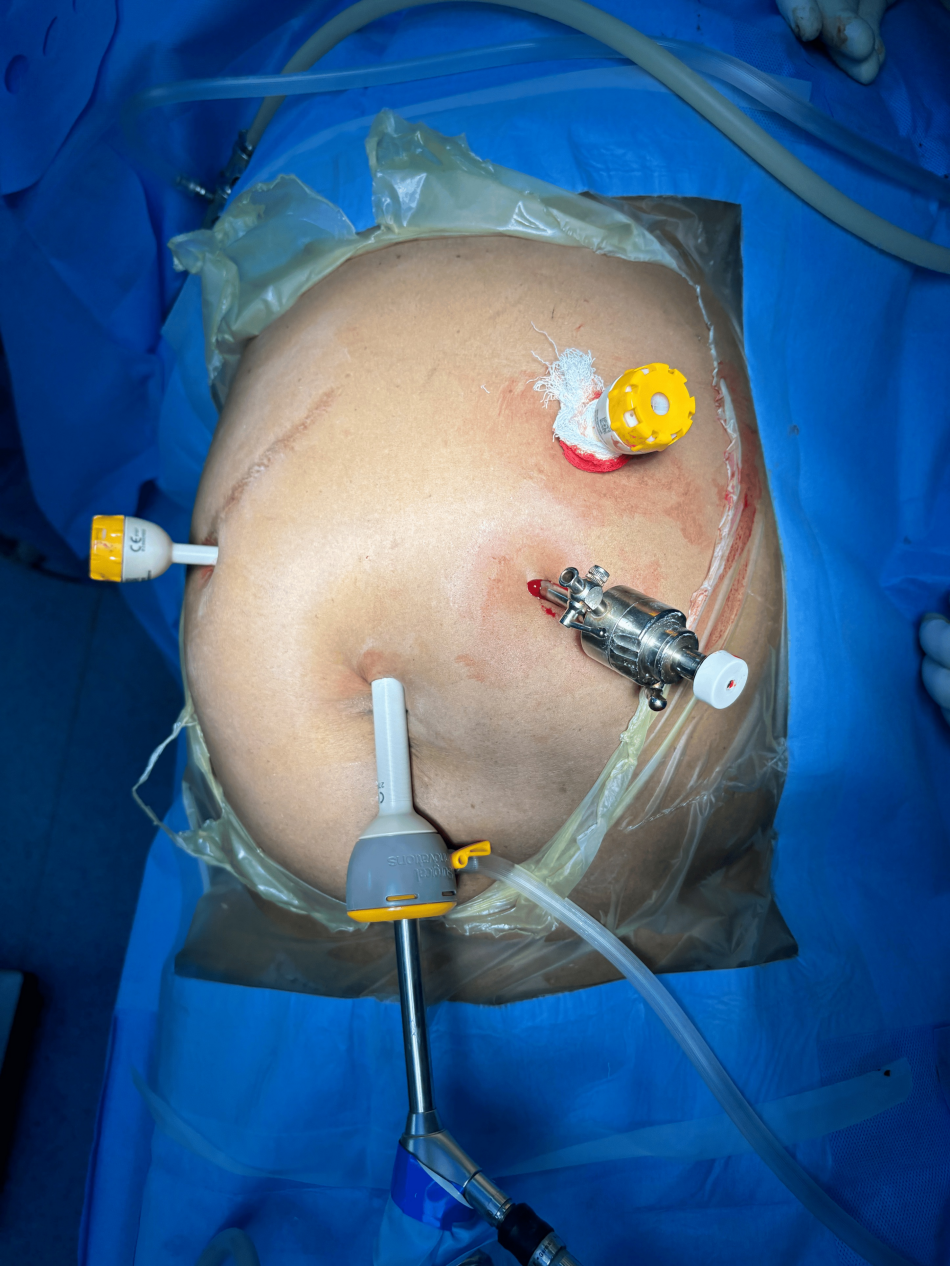
The robotic port placement. Targeted right upper quadrant (RUQ) four-port robotic architecture incorporating a dedicated bedside auxiliary assistant port.

**Figure 6:**
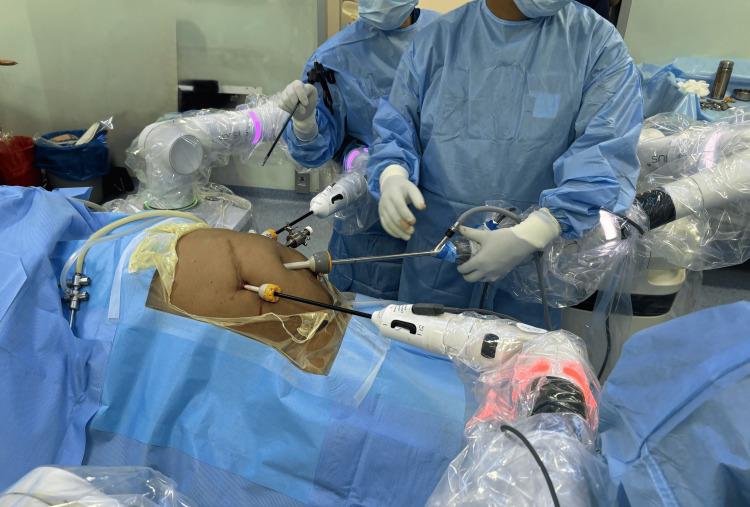
Bedside team orchestration in acute care robotics. Coordinated bedside assistant surgeon workflow utilizing conventional laparoscopic instruments for active retraction and field clearance.

A frequently cited barrier to the adoption of robotic platforms in EGS is the additional setup and docking time required for platform deployment. An optimized operational layout (Figure 4), combined with standardized multi-quadrant port configurations (Figure 5) and a highly coordinated bedside assistant workflow (Figure 6), can significantly mitigate these constraints by structuring clear access perimeters for the surgical team.

The World Society of Emergency Surgery (WSES) position paper identified longer operative duration as a recognized limitation of robotic emergency surgery, particularly during early implementation and in resource-constrained emergency settings [54]. However, several included studies reported low conversion-to-open rates and acceptable perioperative outcomes, suggesting that the potential benefits of enhanced minimally invasive access must be balanced against the additional time required for robotic setup and workflow integration.

Institutional data parse this discrepancy: median total procedure time was 114 minutes, while actual console time was only 78 minutes. This upfront logistical time investment is a calculated trade-off. The additional 20-30 minutes required for setup are clinically justified if they provide the mechanical dexterity needed to definitively complete the case in a minimally invasive manner. Sparing the patient an emergency laparotomy supersedes the premium placed on raw intraoperative speed [55]. The clinical relevance of robotic platforms in EGS may extend beyond operative duration alone. Several included studies reported low conversion-to-open rates in selected complex inflammatory pathologies, suggesting that robotic surgery can facilitate completion of minimally invasive procedures in circumstances where conventional laparoscopy may be technically challenging [12,26]. However, these findings should be interpreted cautiously, as robotic procedures were frequently performed in carefully selected patients and at institutions with established robotic expertise. Consequently, any observed differences in conversion rates and postoperative outcomes may reflect both patient selection and institutional factors in addition to the operative platform itself. Despite these advantages, the broader adoption of robotic surgery in EGS still lags behind elective practice, highlighting the need to address various implementation challenges [8]. For instance, the elevated costs of robotic platforms, coupled with the need for specialized training and dedicated support staff, pose significant institutional hurdles [56].

Comparative Morbidity and Resource Utilization: Complications and Length of Stay

The ultimate clinical and economic arbiters of a surgical platform's value in acute care are postoperative morbidity and hospital LOS. Our EGS synthesis demonstrates that reported complication profiles overlapped substantially between the two minimally invasive modalities, with raw complication incidence ranging from 2.0% to 25.6% across robotic cohorts and from 3.7% to 46.0% across conventional laparoscopic cohorts. However, robotic cohorts consistently report a clinically relevant reduction in LOS (0.7-28.4 days) compared to open surgery, primarily driven by preventing conversions to open abdomens [57]. The methodological quality, however, warrants caution: retrospective registries are subject to severe selection bias, whereby stable patients are triaged to MIS, while critically ill patients default to open surgery. Regarding the unknown horizon, there is a profound absence of randomized controlled trials (RCTs) directly comparing robotic versus laparoscopic outcomes in the acute setting within standardized Enhanced Recovery After Surgery (ERAS) pathways. Furthermore, the economic interplay between the robotic "docking penalty" (increased intraoperative costs) and postoperative savings from a shortened LOS warrants a formal cost-utility analysis [58]. Such an analysis must rigorously account for the substantial acquisition and maintenance costs of robotic systems, which often increase overall procedural costs compared with conventional laparoscopic techniques [23,25].

The Ergonomic Imperative in Acute Care

The ergonomic advantages of the robotic console are frequently dismissed as an elective luxury. In the context of acute care, however, surgeon ergonomics is a critical patient safety metric. Emergency procedures inherently occur out of hours and are performed by surgeons under high physical fatigue and cognitive load. Operating in a frozen, inflamed abdomen via conventional laparoscopy requires unergonomic, sustained physical exertion. The robotic platform offers technical features such as three-dimensional visualization, tremor filtration, articulated instrumentation, and an ergonomically designed surgeon console. Although these characteristics may facilitate dissection and reconstruction in confined operative fields, their independent effects on clinical outcomes remain incompletely defined in the current literature. In the emergency setting, a surgeon’s comfort directly correlates with the stamina required to safely maintain the minimally invasive approach without prematurely converting to open surgery due to frustration or physical exhaustion [6]. This ergonomic benefit not only enhances the surgeon’s performance but also improves patient outcomes by maintaining precision and decision-making throughout demanding emergent procedures. This sustained precision, enabled by robotic systems, may mitigate the risk of surgeon fatigue during complex urgent operations, thereby enhancing overall surgical safety [59].

Systemic Access and Educational Avenues

Despite profound clinical and ergonomic advantages, the adoption of robotics in acute care remains bottlenecked by out-of-hours access, with platforms frequently monopolized by high-yield elective specialties. Interestingly, epidemiological data reveal higher baseline utilization rates in smaller, lower-level trauma centers (19.2 versus 10.5 procedures per 100,000 admissions) [60]. This trend strongly suggests that these decentralized facilities possess the operational flexibility and open scheduling windows needed to organically develop dedicated emergency robotic programs. To safely transition this technology into standard emergency practices, the surgical community must design structured robotic acute care fellowships. These specialized curricula should integrate dual-console intraoperative proctoring with high-fidelity, tissue-quantified emergency simulations to enable trainees to achieve technical mastery over highly inflamed, distorted surgical fields [61]. Furthermore, achieving widespread, scalable adoption requires more than individual surgeon training; it demands a dedicated institutional commitment to overcome persistent logistical barriers, including telerobotic data security, round-the-clock nursing staff availability, and active resource provisioning for 24/7 console coverage [62].

Key Operational Pearls for Deploying Robotics in Acute Care Surgery

Managing the hostile abdomen: Unlike controlled elective fields, the acute abdomen frequently presents with active bleeding or gross enteric contamination. Safe robotic deployment requires immediate visual optimization via aggressive initial suctioning and high-volume irrigation, restricted strictly to hemodynamically stabilized patients outside the hyper-acute damage-control window [8,39].

Fixed multi-quadrant port architecture: Trocar placement must be planned strategically from the outset to establish a comprehensive multi-quadrant abdominal reach. Intraoperative relocation of ports is logistically prohibitive; therefore, baseline layouts must maintain an 8-10 cm inter-trocar distance to prevent external arm collisions while allowing unhindered accessibility from the deep pelvis to the upper quadrants [8].

Open conversion preparedness: The surgical team must maintain constant technical readiness for immediate open conversion. Operating suite setups and bedside cart configurations must preserve a completely clear physical perimeter around the patient, ensuring that rapid manual undocking and emergency midline laparotomy can be completed in under 60 seconds if catastrophic hemorrhage is encountered [8].

The institutional out-of-hours bottleneck: Widespread acute care scalability relies heavily on dedicated institutional infrastructure. Success requires a structured framework that guarantees 24/7 console availability, round-the-clock specialized emergency nursing staff, and rapid technical support teams to handle out-of-hours emergencies [32,62].

Future Perspectives: Telerobotics and Decentralized Acute Care

Telerobotics represents a compelling future application of robotic technology in acute care surgery, potentially allowing expert trauma surgeons to provide real-time operative support to remote or austere environments. However, the feasibility, safety, regulatory considerations, data security, and clinical effectiveness of telerobotic trauma surgery remain largely unexplored and require dedicated prospective evaluation before routine clinical implementation can be considered [62].

Several limitations of the current literature must also be acknowledged when interpreting this synthesis. The available evidence is predominantly retrospective, consisting of administrative database analyses and single-center case series that are inherently subject to selection bias. Furthermore, substantial heterogeneity in patient selection, disease severity classifications, and outcome definitions limits the ability to perform direct comparative assessments across platforms. Finally, the complete absence of prospective RCTs comparing robotic and laparoscopic approaches in the acute care setting highlights the need for well-designed, multicenter studies to define the true comparative effectiveness and cost-utility of this technology.

Strengths and Limitations

The primary strength of this systematic review is the deliberate separation of EGS and visceral trauma into distinct analytical cohorts, thereby minimizing methodological confounding between two fundamentally different acute care populations. Furthermore, by incorporating large national registries, administrative database studies, institutional cohorts, and detailed trauma case reports, this review provides a comprehensive overview of the current applications and clinical boundaries of robotic surgery within acute care settings.

Several limitations should be acknowledged. First, substantial clinical and methodological heterogeneity existed across the included studies. The EGS cohort encompassed a broad spectrum of pathologies, including acute biliary disease, abdominal wall hernias, foregut emergencies, and colorectal pathology, limiting direct comparisons between studies. Consequently, outcomes were synthesized descriptively rather than pooled statistically. Second, the trauma literature remains immature and is dominated by case reports and small case series, with only limited registry-based observational data available. As a result, the trauma findings primarily demonstrate technical feasibility and potential clinical applications rather than comparative effectiveness.

Third, the majority of EGS evidence is derived from retrospective observational studies and large administrative databases, introducing an inherent risk of selection bias. Robotic procedures were frequently performed in carefully selected patients treated at centers with established robotic expertise, whereas physiologically unstable patients or those requiring immediate damage-control intervention were more likely to undergo open surgery. Consequently, observed differences in perioperative outcomes may reflect both patient selection and institutional factors in addition to the operative platform itself. Finally, definitions of "urgent," "emergent," and "semi-acute" intervention varied considerably across the literature, limiting direct comparisons of intervention timing and clinical applicability between studies.

## Conclusions

Robotic-assisted surgery appears to be a feasible minimally invasive approach in selected acute care settings. Within EGS, the available evidence suggests that robotic platforms can be safely applied to complex biliary, hernia, and colorectal emergencies, with several studies reporting low conversion-to-open rates and acceptable perioperative outcomes. However, the predominance of retrospective observational data and the substantial heterogeneity of included pathologies preclude definitive conclusions regarding comparative superiority over conventional laparoscopic approaches. In visceral trauma, the current evidence indicates that robotic surgery is utilized almost exclusively in hemodynamically stable patients following completion of initial resuscitation. Reported applications are concentrated in delayed or semi-acute reconstructions involving anatomically confined regions such as the diaphragm, thoracic cavity, and genitourinary tract. The available literature therefore supports technical feasibility in carefully selected patients rather than routine use during damage-control trauma surgery.

Collectively, the available evidence suggests that the role of robotic surgery in acute care is not universal but rather confined to carefully selected patients and specific clinical scenarios where enhanced visualization and instrument articulation may facilitate minimally invasive management. Future adoption of robotic platforms in acute care will depend upon the generation of higher-quality comparative evidence, improved institutional access, and the development of structured emergency robotic surgery training pathways.

## Appendices

Supplementary appendix 1

Detailed Literature Search Strategy and Database-Specific Search Strings

1. Search framework (PICO)

The systematic search strategy was developed using the PICO (Population, Intervention, Comparison, and Outcome) framework to evaluate the clinical utility of robotic platforms in acute care surgery. As the review addressed two distinct acute care domains, i.e., emergency general surgery (EGS) and visceral trauma, separate search algorithms were applied for each cohort across all databases and subsequently combined during screening and eligibility assessment.

Population (P): Adult patients (≥18 years) presenting with emergency general surgical pathology (e.g., acute biliary disease, incarcerated or strangulated hernias, gastrointestinal perforations, bowel obstruction, and colorectal emergencies) or visceral traumatic injuries requiring urgent, emergent, or delayed operative intervention. Pediatric populations (<18 years) were excluded.

Intervention (I): Robotic-assisted surgical platforms used for operative exploration, resection, repair, or reconstruction.

Comparison (C): Conventional laparoscopic or open surgical approaches where comparative data were available. Non-comparative designs (case reports and case series) were considered eligible within the trauma cohort because of the limited maturity of the evidence base.

Outcomes (O): Primary outcomes included conversion to open surgery, technical feasibility/success, and timing of intervention. Secondary outcomes included operative duration, hospital length of stay, postoperative morbidity, and resource utilization.

2. Search logistics and eligibility limits

Databases searched: PubMed/MEDLINE; Embase; Scopus; Web of Science; and Cochrane Library.

Search period: Database inception through March 2026.

Language restriction: English-language publications only.

Study population: Human subjects only.

Reference management: All retrieved citations were imported into Zotero reference management software (Corporation for Digital Scholarship, Vienna, VA) for duplicate removal, eligibility tagging, and study screening prior to full-text assessment.

3. Database-specific search strategies

A. PubMed/MEDLINE

Searches were performed using a combination of Medical Subject Headings (MeSH) and free-text keywords.

Emergency general surgery (EGS): ("Robotic Surgical Procedures"[Mesh] OR "Robotics"[Mesh] OR robot* OR "robot-assisted" OR "robotic surgery" OR "robotic-assisted surgery") AND ("Emergencies"[Mesh] OR "Emergency Treatment"[Mesh] OR "emergency surgery" OR "acute care surgery" OR "emergency general surgery" OR "acute surgical emergency").

Visceral trauma: ("Robotic Surgical Procedures"[Mesh] OR "Robotics"[Mesh] OR robot* OR "robot-assisted" OR "robotic surgery") AND ("Wounds and Injuries"[Mesh] OR "Abdominal Injuries"[Mesh] OR "Thoracic Injuries"[Mesh] OR trauma OR "trauma surgery" OR "visceral trauma" OR "abdominal trauma").

B. Embase (Elsevier)

Searches were conducted using Emtree subject headings and free-text terminology.

Emergency general surgery (EGS): ('robotic surgical procedure'/exp OR 'robotics'/exp OR robot*:ti,ab,kw OR 'robot-assisted':ti,ab,kw OR 'robotic surgery':ti,ab,kw) AND ('emergency surgery'/exp OR 'acute care surgery':ti,ab,kw OR 'emergency general surgery':ti,ab,kw OR 'acute surgical emergency':ti,ab,kw).

Visceral trauma: ('robotic surgical procedure'/exp OR 'robotics'/exp OR robot*:ti,ab,kw OR 'robot-assisted':ti,ab,kw OR 'robotic surgery':ti,ab,kw) AND ('trauma'/exp OR 'abdominal injury'/exp OR 'thoracic injury'/exp OR 'trauma surgery':ti,ab,kw OR 'visceral trauma':ti,ab,kw OR 'abdominal trauma':ti,ab,kw).

C. Scopus (Elsevier)

Searches were conducted using title, abstract, and keyword fields.

Emergency general surgery (EGS): TITLE-ABS-KEY (robot* OR "robot-assisted" OR "robotic surgery" OR "robotic-assisted surgery") AND TITLE-ABS-KEY ("emergency surgery" OR "acute care surgery" OR "emergency general surgery" OR "acute surgical emergency").

Visceral trauma: TITLE-ABS-KEY (robot* OR "robot-assisted" OR "robotic surgery") AND TITLE-ABS-KEY ("trauma surgery" OR trauma OR "visceral trauma" OR "abdominal trauma").

D. Web of Science (Clarivate)

Topic searches were performed using combinations of robotic surgery and emergency or trauma terminology.

Emergency general surgery (EGS): TS=(robot* AND ("emergency surgery" OR "acute care surgery" OR "emergency general surgery")).

Visceral trauma: TS=(robot* AND ("trauma surgery" OR "visceral trauma" OR "abdominal trauma")).

E. Cochrane Library (Wiley)

Keyword combinations analogous to the PubMed strategy were employed.

Emergency general surgery (EGS): (robot* OR "robot-assisted" OR "robotic surgery") AND ("emergency surgery" OR "acute care surgery" OR "emergency general surgery").

Visceral trauma: (robot* OR "robot-assisted" OR "robotic surgery") AND ("trauma surgery" OR "visceral trauma" OR "abdominal trauma").

Database records identified through the Cochrane Library search were screened; however, no unique primary empirical studies that met the final inclusion criteria were found in this source.

4. Secondary search and cross-referencing procedures

To minimize the risk of missing eligible studies due to indexing limitations or database-specific retrieval bias, supplementary manual search procedures were conducted.

(1) Reference lists of all included primary studies were manually reviewed to identify potentially eligible publications not captured by electronic database searches.

(2) Reference lists of relevant narrative reviews, systematic reviews, meta-analyses, and position papers identified during screening were also examined to identify additional primary studies suitable for inclusion.

(3) Duplicate records identified across databases were removed using Zotero-assisted matching and manual verification prior to title and abstract screening.

(4) All eligible studies identified through secondary searching underwent the same predefined screening and eligibility assessment process as electronically retrieved records.

Supplementary appendix 2

Methodological Quality Assessment and Risk-of-Bias Evaluation

Quality assessment strategy

The methodological quality and risk of bias of all included primary studies were assessed according to study design.

For the EGS cohort, comparative observational studies, retrospective cohort studies, registry investigations, administrative database studies, propensity-matched analyses, and institutional cohort studies were evaluated using the Newcastle-Ottawa Scale (NOS), which assesses methodological quality across three domains: cohort selection, comparability of study groups, and outcome assessment.

For the visceral trauma cohort, study quality was assessed using the Joanna Briggs Institute (JBI) critical appraisal tools. Individual case reports were evaluated using the JBI checklist for case reports; case series were evaluated using the JBI checklist for case series; and observational registry-based studies were evaluated using the appropriate JBI analytical study checklist.

Because of the marked heterogeneity of study designs and the predominance of descriptive trauma literature, quality assessment was performed to characterize the overall strength and limitations of the evidence base rather than to generate pooled quality scores.

Emergency General Surgery (EGS) Cohort

Included study designs: The EGS cohort comprised 22 primary studies, including the following: retrospective cohort studies; registry-based investigations; administrative database analyses; propensity-matched studies; case-matched studies; institutional cohort studies; feasibility studies and case series.

Overall Methodological Quality

The EGS literature demonstrated moderate overall methodological quality. Most studies included clearly defined patient populations, clinically relevant outcomes, and appropriate descriptions of operative techniques. Several large-scale registry and database investigations incorporated statistical adjustment methods, including propensity matching and inverse probability weighting, to reduce confounding.

However, all included EGS studies were observational in nature. Consequently, several inherent limitations were consistently identified: selection bias related to patient allocation; residual confounding despite statistical adjustment; institutional variation in robotic platform access; differences in surgeon experience and learning curves; absence of randomized controlled trials (Table 3).

**Table 3:** Risk-of-bias summary: EGS cohort. EGS: emergency general surgery.

Domain	Assessment
Selection bias	Moderate
Confounding bias	Moderate
Outcome reporting bias	Low–moderate
Attrition bias	Low
External validity	Moderate
Overall certainty of evidence	Moderate

Visceral Trauma Cohort

Included study designs: The visceral trauma cohort comprised 14 primary studies, including individual case reports, small case series, and registry-based observational data.

Overall Methodological Quality

The trauma literature remains an emerging field and is dominated by descriptive evidence. Most publications provided detailed operative descriptions, clearly documented injury patterns, and adequately reported short-term postoperative outcomes.

The principal methodological limitations identified across the trauma literature included small sample sizes, absence of comparator groups, publication bias favoring successful cases, limited long-term follow-up, inability to perform meaningful statistical adjustment, and limited generalizability to broader trauma populations.

The single registry-based study provided additional observational data regarding patient selection and timing of intervention but remained subject to the limitations inherent to retrospective registry analyses (Table 4).

**Table 4:** Risk-of-bias summary: trauma cohort.

Domain	Assessment
Selection bias	High
Confounding bias	High
Publication bias	High
Outcome reporting bias	Moderate
External validity	Low
Overall certainty of evidence	Low

Overall Interpretation of Evidence Quality

The quality assessment demonstrated substantial differences in the maturity of the evidence base between the two acute care domains evaluated.

The EGS literature is supported primarily by comparative observational studies and large-scale registry analyses, permitting descriptive evaluation of clinical outcomes with moderate confidence. In contrast, the visceral trauma literature remains largely exploratory and is supported predominantly by case reports and small case series. Consequently, findings from the trauma cohort should be interpreted primarily as evidence of technical feasibility and proof of concept rather than as definitive evidence of comparative effectiveness.

These findings support the use of robotic surgery in selected EGS applications while highlighting the need for prospective multicenter studies and higher-level comparative evidence to better define its role in visceral trauma.
